# Physical and mechanical cues affecting biomaterial-mediated plasmid DNA delivery: insights into non-viral delivery systems

**DOI:** 10.1186/s43141-021-00194-3

**Published:** 2021-06-17

**Authors:** Valeria Graceffa

**Affiliations:** 1 Cellular Health and Toxicology Research Group (CHAT), Institute of Technology Sligo, Ash Ln, Bellanode, Sligo, Ireland; 2 Department of Life Sciences, Institute of Technology Sligo, Ash Ln, Bellanode, Sligo, Ireland

**Keywords:** Biomaterial-mediated gene delivery, Surface-mediated gene delivery, Non-viral gene delivery systems, Mechanical cues, Extracellular matrix cues, Topographic cues

## Abstract

**Background:**

Whilst traditional strategies to increase transfection efficiency of non-viral systems aimed at modifying the vector or the polyplexes/lipoplexes, biomaterial-mediated gene delivery has recently sparked increased interest. This review aims at discussing biomaterial properties and unravelling underlying mechanisms of action, for biomaterial-mediated gene delivery. DNA internalisation and cytoplasmic transport are initially discussed. DNA immobilisation, encapsulation and surface-mediated gene delivery (SMD), the role of extracellular matrix (ECM) and topographical cues, biomaterial stiffness and mechanical stimulation are finally outlined.

**Main text:**

Endocytic pathways and mechanisms to escape the lysosomal network are highly variable. They depend on cell and DNA complex types but can be diverted using appropriate biomaterials. 3D scaffolds are generally fabricated via DNA immobilisation or encapsulation. Degradation rate and interaction with the vector affect temporal patterns of DNA release and transgene expression. In SMD, DNA is instead coated on 2D surfaces. SMD allows the incorporation of topographical cues, which, by inducing cytoskeletal re-arrangements, modulate DNA endocytosis. Incorporation of ECM mimetics allows cell type-specific transfection, whereas in spite of discordances in terms of optimal loading regimens, it is recognised that mechanical loading facilitates gene transfection. Finally, stiffer 2D substrates enhance DNA internalisation, whereas in 3D scaffolds, the role of stiffness is still dubious.

**Conclusion:**

Although it is recognised that biomaterials allow the creation of tailored non-viral gene delivery systems, there still are many outstanding questions. A better characterisation of endocytic pathways would allow the diversion of cell adhesion processes and cytoskeletal dynamics, in order to increase cellular transfection. Further research on optimal biomaterial mechanical properties, cell ligand density and loading regimens is limited by the fact that such parameters influence a plethora of other different processes (e.g. cellular adhesion, spreading, migration, infiltration, and proliferation, DNA diffusion and release) which may in turn modulate gene delivery. Only a better understanding of these processes may allow the creation of novel robust engineered systems, potentially opening up a whole new area of biomaterial-guided gene delivery for non-viral systems.

## Background

Numerous are the applications of gene therapy and span from the treatment of genetic diseases to the reduction of inflammatory processes [1], tissue engineering [2] and stem cell differentiation [3]. Concomitantly, several challenges and setbacks are still limiting their clinical implementation. In spite of high transfection efficiency, viral transfection systems arise safety concerns, with few deaths being reported during viral-based gene therapy trials [4–6]. On the other hand, non-viral systems cross the plasma membrane barrier, escape lysosomal degradation and are delivered to the nucleus, less efficiently than viral ones [7].

Whilst traditional strategies to increase efficiency of non-viral systems aimed at modifying the vector or optimising the design of polyplexes/lipoplexes, biomaterial-mediated gene delivery has recently sparked increased interest within the scientific community. DNA polyplexes or lipoplexes can be embedded, immobilised or coated on biomaterials. Not only do the biomaterials increase localisation at the desired site, offer a support for cells to grow and a mechanical support [2, 8], but they also directly modulate transfection efficiency. As a matter of fact, cell embedding [9], cell adhesion [10, 11] and migration [12, 13] influence cytoskeletal arrangement and focal adhesions, ultimately affecting DNA internalisation and nuclear transport. Biomaterial-mediated gene delivery systems generally result in a higher transfection [14–16] and even reduced cytotoxicity [15], than traditional ones. For instance, 3D culture systems containing mineral-coated microparticles releasing DNA polyplexes resulted in a higher transfection rate of human mesenchymal stem cells (MSCs), compared to bolus delivery. This was attributed to an increase in macropinocytosis-mediated DNA uptake [17]. Different cell seeding strategies may further modulate transfection efficacy and temporal pattern of transgene expression. For instance, NIH3T3 murine fibroblasts, if embedded into fibrin hydrogels containing Lipofectamine™ (Invitrogen) complexes, were more efficiently transfected, than when seeded on top of the gels. The former approach was also less reliant on biomaterial degradation [15]. Similarly, another study found an initial lower transfection rate, but a prolonged transgene expression, for cells seeded on top of hydrogels, compared to cells encapsulated within the gels [18].

A drawback of many non-viral systems is their inability to integrate plasmid DNA. However, especially in the case of non-cycling cells, which show a reduced tendency to lose episomal DNA [19], episomal maintenance of vectors would be in fact desirable over random integration for toxicity, safety-related concerns and technical complexity [20]. By combining optimal biomaterials with non-viral systems, a sufficient prolonged transgene expression can be achieved. For instance, in immunocompetent murine models [20–24], sustained transgene expression in vivo in the retina was demonstrated for even 6 months [20] and 2 years [25], in the skeletal muscle for at least two [21] and 19 months [24], whereas subcutaneously implanted poly(lactic-co-glycolic acid) (PLGA) scaffolds allowed expression of the transgene for at least 28 weeks [23] and 126 days [22].

However, parameters determining temporal pattern of gene expression and efficacy of transfection are not only limited to the dimensionality and to the cell seeding approach. Interaction between cells, biomaterial and DNA is in fact extremely complex and mechanisms involved are still not fully understood. This review proposes to critically discuss biomaterial properties and to unravel underlying mechanisms of action that need to be considered when designing a biomaterial-mediated gene delivery system. Mechanisms of lipoplex/polyplex internalisation and nuclear delivery are initially discussed, as their understanding allows development of targeted approaches. Differences between DNA immobilisation, encapsulation and surface-mediated delivery (SMD) are then highlighted. The effect of extracellular matrix (ECM) mimetics, biomaterial stiffness and mechanical stimulation is finally outlined.

## Main text

### DNA delivery and trafficking within the cells

Common DNA delivery systems are polyplexes, lipoplexes and nioplexes. The former are composed of polymers, whose positive charge enables binding to DNA molecules and facilitates interaction with cell membrane [26]. Lipoplexes are instead complexes of DNA, cationic and neutral lipids [27]. Specifically, the cationic lipids 1,2-dioleoyl-3-trimethylammonium propane (DOTAP), 1,2-di-O-octadecenyl-3-trimethylammonium propane (DOTMA) are commonly used, along with the neutral dioleoylphosphocholine (DOPC), 1,2-dioleoyl-sn-glycero-3-phosphoethanolamine (DOPE) and cholesterol [28]. Nioplexes are formed by niosomes, which are single-chain vesicles containing both non-ionic surfactants, and cationic lipids interacting with nucleic acids. Their size can vary from 10 to 3000 nm [29, 30].

**Fig. 1:**
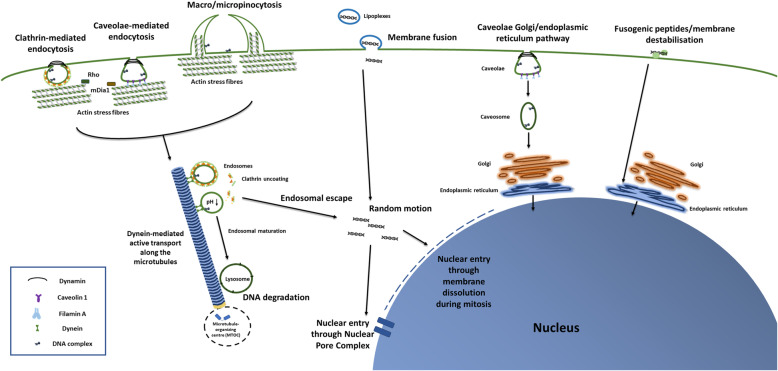
Schematic representation of DNA endocytosis, cytoskeletal trafficking, and nuclear entry. Description of figure from left to right: DNA complexes enter the cells via endocytosis, through clathrin-coated vesicles, through caveolae or through pinocytosis. Their entry is regulated by Rho proteins and mDia1, which control actin dynamics. Dynamin mediates both clathrin-dependent and caveola-dependent endocytosis, whereas Caveolin 1 and Filamin A only mediate caveolae internalisation. Endocytic vesicles are transported by dynein and move along the microtubules. DNA complexes escaping the endocytic trafficking enter the nucleus. Nuclear entry either happens during cellular mitosis or through the Nuclear Pore Complex. A small portion of lipoplexes can enter the cells through direct membrane fusion. Certain caveolar vesicles were instead shown to directly target the Golgi, preventing DNA from reaching the lysosomes. Similarly, despite mechanisms being still obscure, certain CPPs can either fuse with or destabilise the plasma membrane, allowing the DNA to directly reach the Golgi-Endoplasmic Reticulum network, avoiding the lysosomal degradation [32–34].

Polyplexes, lipoplexes and nioplexes do not only act as mere DNA carriers, but play an active role in modulating endocytic pathway, DNA cytoskeletal trafficking and nuclear entry [31]. To be correctly expressed, DNA molecules need to overcome several cellular barriers. The first is the cell membrane, and DNA complexes are generally internalised via clathrin-mediated, caveola-mediated endocytosis, or micro and micropinocytosis (Fig. 1). Formation of both clathrin-coated vesicles and caveolae starts with the invagination of the cell membrane and depends on dynamin activity [32]. Macro and micropinocytosis instead, refer to the endocytosis of liquid materials and involve membrane deformations encircling the liquid. They both share common post-endocytic events [35], but the former refers to vesicles with a diameter bigger than 0.2 μm, the latter to smaller ones [36].

Inhibition of clathrin-mediated route by chlorpromazine [17, 37, 38], of caveolae using methyl-β-cyclodextrin [17], ,genistein [38] or filipin III [37], of micropinocytosis using amiloride or wortmannin [38] decreased transfection efficiency, to a different extent depending on complex type. In general, when cholesterol and DOPE were utilised [33], internalisation of liposomes were cholesterol sensitive [39, 40]. Liposomes containing high amount of DOTAP and DOPC might tend to interact with fluid-phase domains, rich of unsaturated lipids [33]. A small portion of lipoplexes could even enter the cells through direct membrane fusion [41, 42]. Although studies using endocytosis inhibitors provided an initial understanding of internalisation mechanisms, it is noteworthy to mention that common inhibitors may show poor specificity and their inhibitory effects are highly cell line dependant [43].

Dependence of gene transfection to different endocytic pathways also differed as a function of cell type. For instance, polyethylenimine (PEI) polyplexes were exclusively internalised via clathrin-coated vesicles by COS-7 (monkey kidney fibroblast-like cells), but by both clathrin and caveola-dependant routes by HeLa (cervical cancer cells) [37]. In HUH-7 cells (human liver cells), linear PEI was mainly internalised via the clathrin-dependent route, whereas branched PEI by both clathrin-dependent and caveola-dependent routes [37]. On the other hand, in A549 (adenocarcinomic human alveolar basal epithelial cells) [44], in HeLa [44] and COS-7 cells [39], liposomes were internalised by cholesterol-dependent clathrin-mediated endocytosis, whereas in CHO-7 cells through cholesterol-sensitive macropinocytosis [40, 45]. Size of DNA complexes may also affect endocytic pathways, with for instance complexes bigger than 500 nm in diameter being exclusively internalised by caveola-mediated endocytosis [46].

Once internalised, DNA complexes are transported by the cytoskeleton. The actin network regulates the first steps of intracellular uptake [26], whereas microtubules are involved in the subsequent delivery from the endosomes to the lysosomes [47]. The microtubule network transports DNA complexes from the cell periphery towards the microtubule-organising centre (MTOC) (Fig. 1) [48]. Central in this context is the role of Rho, a family of small GTPases, localised at plasma membrane and at the endosomes, and associated with proteins central to actin dynamics (such as the Diaphanous-related formin-1 or mDia1) [49]. Rho proteins mediate the assembly and disassembly of actin stress fibres and microtubules, regulating vesicular transport [50].

Naked DNA delivery methods (e.g. electroporation or nude DNA injection/transfection) may not rely on endocytosis, but do rely on cytoskeletal transport [51]. For instance, after electroporation, actin patches colocalised with DNA at the plasma membrane, and disruption of microfilaments (i.e. polymers of actin) reduced DNA internalisation in CHO-7 cells [52]. Subsequent movement of naked DNA along microtubules was mediated by dynein [51], and followed the classical endosomal-lysosomal route [53].

Only few DNA molecules escape the endosomes before lysosomal degradation (Fig. 1). Disruption of actin filaments [54] and of the endosomal membrane [55] facilitated their escape, eventually increasing transfection of the human T leukaemia cell line (Jurkat) through carbonate apatite particles [54] and of the cell line A549 through liposomes [55].

Among mechanisms to escape endosomes, lipoplexes can directly fuse with endosome membranes, a process which is enhanced by the lipid DOPE [28, 33]. Despite the underlying mechanism being unclear, ability of Lipofectamine™ to avoid active intracellular transport along cytoskeleton is perhaps responsible of its notoriously high transfection efficiency. A study showed indeed that, as opposed to lipoplexes composed of DOTAP and DOPC, Lipofectamine™ exclusively moved within the cytoplasm by free Brownian diffusion [56]. Charge-reversal amphiphiles 1 are also able to escape the lysosomes: the terminal ester of the lipid is hydrolysed, its charge is reversed from + 1 to − 1 and thereby the DNA is released and reaches the cytoplasm [45].

On the other hand, polyplexes with high buffering capacity such as PEI and polyamidoamine (PAMAM) exploit the so called ‘proton sponge effect’, to escape endosomes [57–59]. This is a phenomenon whereby, when endosomal pH lowers, cationic polymers become protonated, causing diffusion of water into the endosome. Eventually, the osmotic pressure makes the endosome swell. This—combined with the swelling of the polymer itself (due to internal electrostatic repulsion of protonated amine groups)—is sufficient to disrupt the membrane, resulting in the escape of nucleic acid into the cytoplasm [60]. Proton sponge can be enhanced by chemical modification of the polymer [61, 62]. Furthermore, highly charged polyplexes (e.g. PEI) can closely interact with the endosomal membrane, induce the formation of pores and destabilise the lipid bilayer. This membrane destabilisation would assist the proton sponge effect [60]. Nevertheless, the proton sponge effect is still not well understood, nor is it clear to which extend it contributes to lysosomal escape [63]. It was for instance hypothesised that cationic polymers cause an influx of protons and of chloride ions [64, 65]: this however, was disproved by experimental data [66, 67]. Similarly, the addition of ammine groups to poly(2-dimethylaminoethyl)-methacrylate) (pDMAEMA)—which increases buffer capacity and theoretically enhances the proton sponge effect—decreased in fact transfection efficiency [68]. On the contrary, acetylation of PEI (which decreases buffer capacity), enhanced gene delivery [69].

Certain cell-penetrating peptides (CPPs) allow endosomal escape, by inducing budding of vesicles from the endosomal membranes, which collapse in the cytoplasm [70].

Once in the cytoplasm, DNA complexes follow a random motion [33] and finally enter the nucleus either passively, during cell division [59] or via the nuclear pore complexes (NPC) [71]. Efficiency of the former mainly depends on the cell cycle stage at the time of transfection [72]. Attempts to improve the inclusion of DNA in the nuclei of the daughter cells relied on either the addition of targeting peptides which bind chromatin or on the incorporation of phosphorylation responsive peptides, which specifically release the DNA during mitosis [73]. However, efficiency of such systems is still controversial [74].

On the other hand, despite the exact mechanism for translocation through the NPC being still largely obscure [71], efficiency of NPC-mediated internalisation can be enhanced, by optimising composition of polyplexes/lipoplexes. For instance, when complexes were injected into cell cytoplasm, PEI, and to a lesser extent polylysine (PLL), increased nuclear transport, compared to cationic lipids [75]. Similarly, in bone marrow mesenchymal stem cells (BMSCs), in spite of a lower cytoplasmic uptake, PEI polyplexes showed a higher percentage of nuclear uptake, compared to PLL [76]. In artificial *Xenopus laevis* nuclei, the size and the charge of PEG nanocomplexes were also proven to affect nuclear internalisation rate [77]. Similarly, after cytoplasmic injection, the supercoiled plasmid DNA form more efficiently reached the perinuclear region, than the relaxed open circular and the linearised forms [78]. Furthermore, some studies attached a peptidic nuclear localisation signal (NLS)—which enables importin-mediated nuclear transport—either to the polymeric carrier, or to the DNA molecule. This approach may increase nuclear localisation: however, it did not markedly increase transgene expression. This probably happened, because the NLS blocked the transcription of the reporter gene or it induced aggregation of the DNA with cellular structures [71]. Among systems mimicking viral entry systems, addition of the protein transduction domain (PTD) of the Tat protein of the HIV also facilitated DNA entering to the nucleus [79, 80].

It is worth mentioning the existence of an alternative internalisation route, mediated by caveolae, whereby the DNA complexes do not reach the endocytic network, but the Golgi [44, 81–83]. Addition of the histone H3 peptide tail in DNA polyplexes enhanced this route [83]. However, no consensus has been reached in the scientific community on whether all [81, 84] or only specialised subsets [85, 86] of caveola-derived endosomes follow this route. Similarly, despite their mechanisms of internalisation being still largely obscure [87, 88], certain CPPs specifically target the endoplasmic reticulum (ER) close to the nucleus. For instance, lipoplexes, conjugated with the pardaxin (i.e. a single polypeptide chain composed of 33 amino acids) avoided the endosomal network [57, 89]. From the ER, DNA can easily enter the nucleus [57], as the membranes of the two organelles are continuous [90].

Understanding the processes regulating gene transport along the cytoplasm is fundamental to develop tailored gene delivery strategies. For instance, strategies enhancing caveola-mediated endocytosis may avoid the lysosomal degradation, increasing transfection yield [81]. Endocytic pathways differs as a function of dimensionality, with 3D systems being generally associated with increase in caveola-mediated endocytosis. In one study, jetPEI® polyplexes (Polyplus-transfection® SA) were internalised via caveolae and micropinocytosis by cells grown on microporous annealed particle (MAP) 3D hydrogels, but exclusively via clathrin-endocytic pathways, by cells seeded on plastic tissue dishes (2D cultures). Such differences were attributable to activation of distinct Rho proteins, with for instance Rac and Cdc42 being more integral to gene transfer, when cells were grown on the hydrogels [91]. Also addition of collagen-mimetic peptides to PEI polyplexes immobilised on collagen scaffolds, activated a specific endocytic pathway, mediated by the collagen, and involving the caveolae [92]. Furthermore, increase in the expression of RhoA, in caveola-mediated endocytosis and in polyplex-mediated transfection efficiency was reported for rat adipose-derived stem cells (ADSCs) cultured on hyaluronic acid (HA)-chitosan surfaces (and to a lesser extent on unconjugated chitosan surfaces), compared to cells on tissue plastic dishes [93].

Processes altering the cytoskeleton, such as cell encapsulation, cell spreading, adhesion, or mechanical loading further mediate DNA cellular trafficking. Understanding these mechanisms can allow to divert cell adhesion processes and cytoskeletal dynamics, in an effort to increase transgene expression [26]. Strategies described and their outcomes are discussed in the following paragraphs.

### DNA immobilisation and encapsulation in 3D scaffolds

**Fig. 2:**
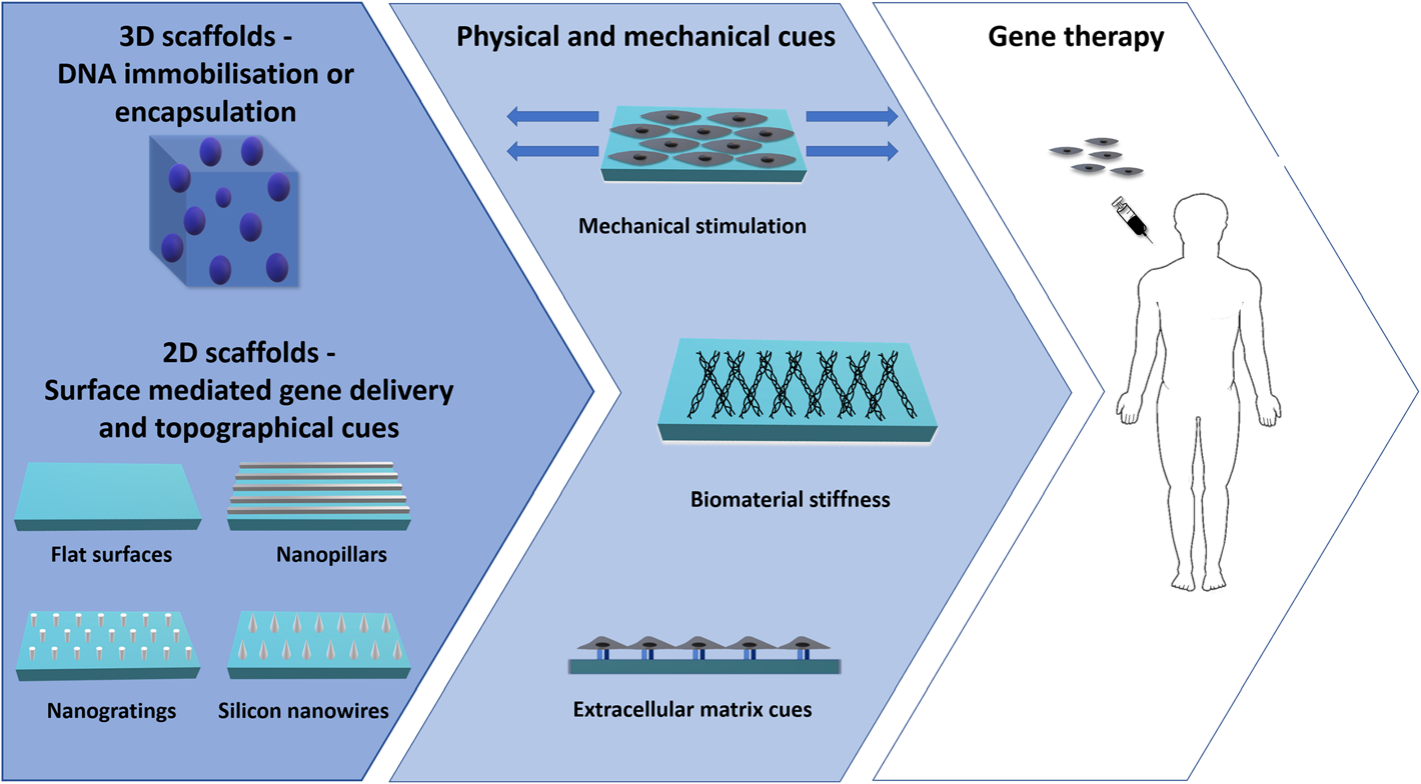
Schematic representation of biomaterial-mediated gene delivery systems. DNA can be incapsulated/ immobilised into 3D biomaterials or coated on 2D substrates (SMD). 2D materials may be flat, or contain topographical cues, such as nanopillars, nanogratings or silicon nanowires. Physical and mechanical cues (i.e. mechanical stimulation, optimal biomaterial stiffness and ECM cues) may further enhance transfection efficiency. Addition of ECM mimetics may even allow cell type-specific transfection

Both 2D and 3D biomaterials are largely used for gene delivery purposes. 3D scaffolds are generally fabricated thorough DNA encapsulation or immobilisation, whereas SMD utilises DNA-coated 2D materials. (Fig. 2) Addition of physical and mechanical cues (e.g. mild mechanical loading, optimal biomaterial stiffness and extracellular matrix cues) may further enhance gene delivery. (Fig. 2) With respect to the fabrication of 3D scaffolds, encapsulation refers to the entrapment of DNA complexes within the biomaterials, whereas immobilisation requires interaction between the vectors and the biomaterial. In the latter case, since the DNA is physically attached, its mobility is reduced [23, 94]. DNA is thereby released, gradually with biomaterial degradation/cellular infiltration [15, 95], or—depending on fabrication parameters—in response to specific stimuli (e.g. variation in the pH, salt concentration or electrochemical and thermal triggers) [96].

For immobilisation, the 3D biomaterial can either be first synthesised, and DNA only added in the final steps (to avoid exposure to potential harsh processing steps) (i.e. surface immobilisation) [97, 98] or can be mixed with the DNA prior to polymerisation [92].

Microporous scaffolds, with pores on the order of tens to hundreds of microns, have been largely used [97, 99–102], and compared to non-porous biomaterials they facilitated cell infiltration [99, 100, 102] DNA diffusion and release [99], eventually increasing [100, 101] and prolonging [100] transgene expression. Porous material may also result in more biodegradable, than non-porous ones [99, 101]. Different DNA encapsulation techniques on oligo (polyethylene glycol) fumarate (OPF) hydrogels [103], alginate [104], polyethylene glycol **(**PEG) [105, 106], hyaluronic acid [99], fibrin [107], PLGA [23, 108] or gelatin [109] hydrogels were described. Similarly, immobilisation on polyacrylamide [110], cationised gelatin hydrogels [111], poly(beta-amino ester)(PBE)/PLGA microparticles [112] and PLGA biomaterials [113], on polydopamine (PD) conjugated materials [114] and of collagen-mimetic peptides – conjugated polyplexes [115] were performed. Enzyme-responsive gene delivery systems furthermore allowed temporal control of DNA release and were fabricated by incorporating the matrix metalloproteinase (MMP) susceptible peptides GPQGIWGQ [116] and the GCRD-GPQGIWGQDRCG [117, 118] into an Au surface [116], into a breath figure porous structures [117] or in PEG hydrogels [118]. By using similar approaches, pH-sensitive [119, 120], thermosensitive [120, 121] or even ultrasound-activated [122, 123], visible-light assisted [124], photothermal-assisted [125] gene delivery systems were developed.

The main parameters affecting nucleic acid release are the biomaterial degradation rate, the size and amount of the DNA complexes and their interaction with the biomaterial.

Not only does degradation rate influence cell infiltration, but it also modulates temporal pattern of DNA release [98, 126]. Biomaterials degrade over time, gradually releasing the DNA [16, 126, 127], with totality of complexes being released after complete degradation [15]. The peak in the expression of the transgene is generally observed after complete degradation of the biomaterial, yet transgene expression in vitro [128] and in vivo [23] was maintained after several weeks. The amount of time needed to achieve complete degradation obviously varied depending on fabrication method and eventual crosslinking [127–129]. Slow degradation rate may reduce transfection efficiency at earlier timepoint [15], but extend temporal pattern or DNA release [130]. For instance, PLGA scaffolds fabricated using supercritical CO_2_ gas were more stable than that prepared with high-pressure CO_2_. They allowed the release of 50% of DNA after 60 days, and in vivo transgene expression was detected even at longer timepoints [130].

On the other hand, the size of DNA complexes affects their diffusion within the biomaterial. For instance, one study prepared 4 KDa PEI polyplexes with an average size of 142 nm, 40-kDa PEI polyplexes with a size of 243 nm and Lipofectamine™ lipoplexes with a size of 342 nm. The larger the complexes, the slower the diffusion within the gels [109]. Similarly, naked DNA molecules are better diffused within HA/PEG hydrogels, compared to DNA/PEI complexes, probably due to their smaller hydrodynamic diameter [131]. The amount of plasmid loaded also plays a key role in regulating transgene expression. PLGA matrices loaded with 1.6 μg/μL showed higher transfection efficiency, than that loaded with 1 μg/μL. After subcutaneous implantation into mice, transgene expression was detectable even after 126 days [22]. Similarly, fibrin hydrogels loaded with 2 μg of DNA showed higher transgene expression, compared to hydrogels loaded with 0.1 μg [18]. Yet, loading high DNA concentration can be technically difficult, due to its tendency to aggregate [132], which may even compromise cellular transfection [18]. Addition of neutral saccharides (e.g. sucrose) [105], polysaccharides (e.g. agarose) [107], PEG-modification of polyplexes [106], optimisation of physical properties (size, polydispersity, zeta potential) of complexes [126], can mitigate charge-charge interactions, reducing aggregation tendency. Surface immobilisation could also mitigate polyplex aggregation tendency. One study fabricated HA hydrogel, where PEI/DNA polyplexes were either encapsulated (the complexes were mixed to polymers, prior to polymerisation) or immobilised (DNA complexes were bound to polymerised hydrogels, via electrostatic interaction). The latter resulted in a more homogenous distribution of the plasmid and led to higher transgene expression levels [106]. Similarly, compared to encapsulation, surface immobilisation of PEI complexes in fibrin hydrogels led to a higher transfection and to a faster release. Through surface immobilisation, the DNA was homogeneously exposed to the surface of the biomaterial and was easily accessible by the cells. After encapsulation, the DNA was distributed instead through the gels in larger and possibly aggregated structures. Its exposure was dependent on cell-mediated degradation [127]. However, it is noteworthy to mention a reduction in cellular viability at early timepoints, for surface immobilisation, compared to encapsulation [127].

Finally, enhancing interactions between biomaterial and DNA complex could increase the extent and duration of gene transfer. For instance, incorporation of collagen-mimetic peptides into PEI polyplexes, by increasing interaction with carrier collagen hydrogels, prolonged DNA release [92]. In one study, PLGA microspheres were surface-modified with PEI, PLL, poly(allylamine hydrochloride) (PAH), polydiallyldimethylammonium (PDDA), or PD. [133] The latter—by increasing strength of immobilisation [114]—prolonged the pattern of DNA release from 5 days (of unmodified control microspheres) to 15 days [133].

By understanding how each of these parameters affects cellular transfection, temporal patterning of transgene expression can be tailored, eventually meeting the demands of various applications.

### Surface-mediated gene delivery

SMD is an emerging approach, relying on 2D surfaces, whereby DNA complexes are immobilised to a substrate via covalent attachment or non-specific adsorption [134]. Not only does SMD allow to better control localisation of transfected cells, but—by optimising surface topography—it may also increase cell receptivity to transfection. (Fig. 2). When performed on tissue plastic dishes, SMD generally requires a pre-coating with serum [27, 105] or proteins [135], to mediate nucleic acid immobilisation. Alternatively, lipoplexes/polyplexes containing functional groups that are complementary to the substrate can be used [135]. Different substrates, such as alginate/polycaprolactone fibers [136], polydopamine-coated glass substrates [137], PEI [138], PLGA fibres [113] and polydimethylsiloxane (PDMS) films [139], were used.

The efficacy of SMD has been largely debated. One study compared SMD (serum-coated tissue culture polystyrene), with bolus delivery of PEI polyplexes. One day after transfection, the transfection efficiency of SMD was lower [27]. Another study using Lipofectamine™ lipoplexes showed instead similar transfection efficiency between SMD and bolus delivery [27]. However, it is noteworthy to mention that whilst bolus delivery leads to a peak in gene transfection, when conjugated to biomaterials, DNA complexes are gradually released. Thereby lower amounts may be available at early timepoints, but higher transfection yield can be achieved at longer timepoints [109].

On the other hand, PEI polyplexes, incorporated at the surface of electrospun graphene oxide-incorporated PLGA nanofibrous mat, led to a higher transfection rate of human embryonic kidney cells (HEK293) and human umbilical cord-derived MSCs, compared to bolus delivery [113]. Despite a high biological variability, optimisation of a combination of ECM mimetic peptides and polysaccharides [140] could further increase transfection efficiency. Similarly, as discussed in the next paragraph, incorporation of topographical patterning may modulate DNA endocytosis and cytoskeletal trafficking.

### Surface topography

2D surfaces offer the possibility to incorporate topographic cues, which are generally dominant concerning cells spreading and morphology, over biomaterial composition [141]. For instance, human MSCs cultured on 350-nm gratings showed decreased expression of integrin subunits *α*2, *α*V,*β*2, *β*3 and *β*4 and exhibited an aligned actin cytoskeleton, compared to unpatterned controls [142]. Human amniotic membrane-derived mesenchymal stem cells (hAM-MSCs) and mouse embryonic stem cells (mESCs) showed strong alignment on deep grooves and inhibition of spreading on nanopillars [143]. Topographical cues modulated focal adhesion kinase (FAK) activity and thereby the RhoA phosphorylation level. This was proven to mediate actin fibre arrangement and to activate different signalling pathways, including the mitogen-activated protein kinase (MAPK) cascade [144]. Lipid films with different topologies (i.e. 3D-bicontinuous cubic, 2D-inverted hexagonal, or 1D-lamellar nanostructures) also led to a distinct transfection efficiency [145]. (Table 1) Among different morphological changes induced by substrate topography (i.e. increase in cell spreading, change in nuclear volume, focal adhesions size and area and cellular deformation), the nuclear volume most closely correlated with transfection efficiency [139].

**Table 1:** Surface topography and gene delivery. Research papers assessing the effect of different surface topographies on surface-mediated non-viral gene delivery

Biomaterial	Cell type	Transfection system	Change in transfection efficiency	Ref.
Coverslips coated with a lipid film, having 3D-bicontinuous cubic, 2D-inverted hexagonal, or 1D-lamellar nanostructures	HeLa-Luc cell line, stably expressing the luciferase	siRNA directly loaded into the lipid film	Luciferase activity (luminescence/mg protein) was 4 × 10^5^ in the untreated control. Highest gene silencing efficacy was for 3D-bicontinuous phase (luciferase activity ∼ 1.5. × 10^5^). In 2D-hexagonal and 1D-lamellar phases, luciferase activity was almost 2.5 × 10^5^. Bolus delivery (Lipofectamine™) showed gene silencing efficacy similar to that of 3D-bicontinuous phase.	[145]
Micropatterns with different diameter and aspect ratio	Human MSCs	Cationic polyplexes	Highest transfection efficiency was 20%, for ellipses with aspect ratio 8:1 and surface area 80 μm. Lowest transfection efficiency was 2%, for circular micropattern with a diameter of 20 μm.	[146]
Nanogrooved and nanopillar surfaces with different width/diameter and height	Normal human lung fibroblasts	Lipofectamine™ 2000	Highest % of transfection was 50%, in grooves 500 nm in width and 150 nm in height and in nanopillars with a diameter of 500 nm and a width of 150 nm. In flat surfaces, % transfection was ∼ 30%	[139]
Nanogrooved surfaces with different deep and depth	C2C12 skeletal myoblasts	jetPEI® (Polyplus, US)	Decrease of ∼ 73% in transfection efficiency, on nanogrooved patterns of 400 nm groove width and 400 nm depth, compared to flat surfaces. Decrease of ∼ 90% on nanogrooved pattern of 800 nm groove width and 500 nm depth, compared to flat surfaces.	[147]
Nanogratins and nanopillar surfaces	Human MSCs	Lipofectamine™ 2000	Highest % transfection was at 3.3%, for cells on grooves of 250 nm width. In flat surfaces, % transfection was 1.8%	[148]
SiNWs	Jurkat, L1.2 and GPE86	Naked DNA	% of transfection was ∼ 20%, 20% and 5% in GPE86, L1.2 and Jurkat respectively. % transfection was between 0% and 1%, in flat surfaces for all cell types.	[149]
SiNWs with different heights	Human dental pulp stem cells	Naked DNA	% transfection was ∼ 90% for SiNW with heights of 1.2 and 3.5 μm. % transfection was less than 10% for SiNW with heights of 0.4 μm and 6.3 μm.	[150]
SiNWs	Mouse embryonic stem cells	Naked DNA	Transfection efficiency lower than 1%.	[151]
Ethanolamine-functionalised SiNWs	HeLa	Naked DNA	Luciferase expression (luminescence/mg protein) was 10^6^ in ethanolamine-functionalised siNWs, compared to 10^4^ in non-functionalised siNWs	[152]
Nanopillars of different diameter	C2C12 skeletal myoblasts	jetPRIME® or Lipofectamine™	For jetPRIME®, luciferase expression (luminescence/mg protein) was ∼ 8 × 10^3^ for pillars with a diameter of 1000 nm, whereas in flat surfaces it was 3 × 10^3^ For Lipofetamine™, luciferase expression was 1 × 10^3^ in nanopillars with a diameter 1000 nm and 5 × 10^3^ in flat surfaces.	[153]

In spite of few discordances on how dimensionality regulates cytoskeletal arrangements, it is recognised that a certain degree of cell spreading enhances transfection. (Table 1) Compared to unpatterned substrates, micropatterns facilitating either spreading or elongation of MSCs promoted gene delivery, by enhancing the uptake of the cationic complexes [146]. However, extremely deep nanotopographies may decrease cell adhesion, and consequently cell spreading and nuclear volume [139, 154–156], ultimately jeopardising cellular transfection [139]. C2C12 skeletal myoblasts, if grown on deep nanogrooved surfaces (400-nm and 800-nm depth), showed cytoskeletal stretching, nucleus elongation and reduced nuclear volume. They also were less efficiently transfected by PEI polyplexes, compared to superficial patterns (50 nm). Disruption of F-actin organisation restored nuclear morphology and transgene expression [147]. Similarly, fibroblasts on deep nanogratings and nanopillars (560 nm in heights), showed smaller nuclear volumes and a reduced transfection rate, compared to cells on superficial patterns (150 nm in height) [139]. On the other hand, certain topographic cues can reduce cellular adhesion and spreading, ultimately compromising transfection efficiency. For instance, when human MSCs were seeded on poly(methyl methacrylate) (PMMA) with nanopillars (250 nm in height) and micropillar (2 μm in height), intracellular actin-rich rings outlining the portion of the substrates in contact with the cells was visible. Yet, no such actin-rich regions were seen on nanograting topography (250 nm in height). The latter topography also correlated with the lowest transgene expression (Lipofectamine™) and FITC-dextran uptake [148].

A recently emerging application of SMD is the silicon nanowires (siNWAs). These penetrate the cells, without perturbing their main functions, and release surface-bound DNA complexes, directly inside the cytoplasm [157]. Compared to flat silicon substrates, siNWAs induced morphological changes in the cytoplasm and nuclei, eventually promoting endocytosis in different cell lines, including an immortalised human T lymphocyte cell line (Jurkat), the cellosaurus cell line L1.2 and the murine fibroblast cell line GPE86 [149]. Transfection efficiency was governed by the nanowire geometry (in terms of diameter, length and density of the nanowires), but also varied according to cell type, with for instance HeLa cells showing significantly lower transfection efficiency (7-9%), compared to HEK293 (85-86%), human primary fibroblasts (61%) and dental pulp stem cells (85–88%) [150]. In murine embryonic stem cells-derived cardiac myocytes, reported transfection efficiency was even lower than 1% [151]. Yet, transfection could be increased by surface modification with high-molecular-weight branched PEI [158], or via chemical modification of the siNWAs (e.g. ethanolamine functionalisation of siNWAs) [152] and of the polyplexes (e.g. addition of low concentration of Zn^2+^ on calcium nanoparticles) [152]. Furthermore, one study demonstrated a dependence of SMD-mediated transfection on DNA complex type. This study prepared nanopillars with different diameters (200–1000 nm) and depths (200 or 400 nm), where cells were seeded and transfected with either the polyplex jetPRIME® or Lipofectamine™. It identified two main cell cytoskeletal morphologies after transfection, based on the presence or absence of a perinuclear actin cap (pnAC) in the F-actin. Compared to cells on flat surfaces or on shallow nanopillars, cells on the nanopillars were smaller, less spread, had shorter F-actin filaments, and a lower percentage of pnAC. They showed a significantly lower transfection efficiency with Lipofectamine™, but a higher transfection rate with jetPRIME® [153]. However, as mechanisms of internalisation and nuclear transport of jetPRIME® polyplexes are not known, the difference in transfection efficiency is difficult to explain.

### ECM cues

Early steps of transfection require non-specific binding of cationic DNA complexes with negatively charged molecules at the cell surface, such as the heparan sulphate proteoglycans [26]. Despite this ability being traditionally considered exclusive of viruses, by either modifying composition of complexes [159] or of carrier biomaterials [160–163], it is possible to selectively transfect certain cell types [1]. With respect to modifications on DNA complexes, conjugation with HA facilitated transfection of cells overexpressing the CD44 [28], conjugation of niosomes with transferrin allowed their internalised by transferrin receptor-mediated endocytosis [164], whilst coating of DNA polyplexes with poly-γ glutamic acid (γ-PGA) allowed specific binding to the tumour-associated gamma-glutamyl transpeptidase (GGT) [165]. DNA nanoplexes containing heparin or folic acid PEI derivatives in vitro led to higher transfection efficiency and lower toxicity compared to unmodified PEI [166]. Electrostatic association of nanocrystals of carbonate apatite with fibronectin and/or E-cadherin-Fc accelerated transgene delivery in a human T leukaemia cell line (Jurkat) [54]. Furthermore, one study prepared folate-decorated triblock copolymer delivering siRNA [167], and showed selective transfection of the cell line SKOV-3 cells overexpressing the folate receptor-α (FRα), over the cell line A549, which only had a basal FRα expression. Similarly, after the addition of a N-acetylglucosamine ligand to polymeric nanoparticles, vimentin-expressing cells were more efficiently transfected compared to non-expressing cells, due to a specific polyplex receptor-mediated endocytosis [168].

On the other hand, ECM-derived adhesion peptides, protein fragments, or native proteins can be conjugated to biomaterials, promoting cellular adhesion, and ultimately gene delivery. Different peptides have been used, and some of them may even allow selective transfection of cells expressing specific integrins/membrane receptors (Table 2). The tri-amino acid sequence arginine-glycine-aspartate (RGD), naturally found in collagen and fibronectin and recognised by nearly half of the integrins [184] has been widely utilised (Table 3). In NIH/3T3 cells, Ti substrates with a poly(acrylic) acid (PAA) brush, modified with RGD, showed the highest transfection efficiency, compared to flat Ti surfaces, to PAA brushes modified with a control peptide (RGE) and to unmodified PAA [163]. TransFast™-mediated transfection of NIH3T3 murine fibroblasts was increased by conjugation of PEG hydrogels with RGD peptides [162] and jetPEI® polyplex-mediated transfection of human dermal fibroblasts was increased by conjugation of MAP hydrogels with RGD peptides [185].

**Table 2:** ECM-derived cell adhesion peptides. Most used peptides derived from ECM proteins and targeting specific cell types/cell receptors

Peptide	Specificity
Modified fibronectin (either a Leu-Pro point mutation at position 1408 (9*10) or a labile 4 × Gly linker (9(4G)10) between the ninth and tenth domain domains	Integrin α3/α5β1 or αvβ3 [169]
RGDS sequence from fibronectin	Integrin αvβ3 [170]
REDV sequence from fibronectin	Integrin α4β1 of endothelial cells [171]
LDV sequence from fibronectin	Integrin α4β1 [172]
cRGDfK, cRGDyK and RGDC4 cACRGDMFGCA cyclic peptides from collagen	Integrins αvβ3 [173] and αvβ5 [84]
GGYGGGP(GPP)5GFOGER(GPP)5GPC sequence from collagen	Integrin α2β1, which is expressed by osteoblasts and MSCs during osteogenesis [174]
GFOGER sequence from collagen	Collagen receptors (integrins α1β1, α2β1, α10β1 and α11β1) [174–177]
PDGEA sequence from collagen type I	α2β1 in osteoblasts [178]
PGRGDS sequence from osteopontin	αvβ3 in osteoblasts [178]
DFKLFAVYIKYR-GGC (C16Y) sequence from the mouse laminin	Integrins αvβ3 and α5β1 [179], typical of endothelial cells
IKVAV sequence from laminin	Integrin β1 [180] (α3β1 and α6β1 [84] and α4β1) [181], with high affinity for neuronal cells
RKRLQVQLSIRT (AG73) sequence from laminin	Syndecan-2 [182]
VAPG sequence from elastin	It binds to smooth muscle cells and it is not specific to integrins [183]
RRETAWA synthetic peptide	Integrin α5β1 [181]

**Table 3:** ECM cues and gene delivery. Research papers assessing the effect of ECM cues on biomaterial-mediated gene delivery

Biomaterial	Cell type	Transfection system	Change in transfection efficiency	Ref.
Titanium substrates, modified with RGD-functionalised PAA brushes (N/P ratio of 20)	NIH3T3 murine fibroblasts	PEI polyplexes	In RGD-functionalised PAA brushes, luciferase expression (luminescence/mg protein) was 2.5 × 10^8^. In non-functionalised PAA brushes, it was ∼ 7 × 10^6^.	[163]
Fibrin hydrogels functionalised with PEG-RGD peptides	HT-1080 human fibrosarcoma cell line and NIH3T3 murine fibroblasts	Lipoplexes	For HT-1080, in RGD-functionalised hydrogels, luciferase expression (luminescence/well/day) was ∼ 10^4^, whereas in non-functionalised hydrogels it was ∼ 10^3^. For NIH3T3, in RGD-functionalised hydrogels, luciferase expression (luminescence/well/day) was ∼ 10^4^, whereas in non-functionalised hydrogels it was less than 10^1^.	[162]
Collagen-I-alginate hydrogels, with different collagen: alginate ratios	Human BMSCs	3D-FectIN™ (OZ Biosciences)	Decreasing the collagen: alginate ratio from 1:1 to 1:2 decreased transgene expression 1000-fold.	[14]
MAP hyaluronic acid hydrogels functionalised with RGD peptides.	Human dermal fibroblasts	jetPEI®	High RGD clustering ratio (500 and 100 μM), resulted in a cumulative luciferase expression (luminescence) higher than 2 × 10^7^. Low RGD clustering ratio (100 and 250 μM) resulted in a cumulative luciferase expression of 1 × 10^7^.	[185]
Collagen I, fibronectin, laminin, collagen IV, vitronectin or ECM gel coated plates	Murine MSCs	PEI polyplexes	Highest luciferase expression (luminescence/mg protein) was 7 × 10^8^, in fibronectin-coated plates. Lowest luciferase expression was 2 × 10^6^ in collagen I-coated plates. In uncoated plates, luciferase expression was 4 × 10^7^.	[160]
Denatured or native collagen–PLGA composite vascular stents	A10 murine vascular smooth muscle cell line	Lipofectamine™	Number of transfected cells per 200x field was ∼ 1 for native collagen and almost 20 for denatured collagen. Supplementation of tenascin-C in native collagen substrates increased transfection, with number of transfected cells per 200x field being ∼ 14.	[186]
Fibrin hydrogels, prepared with different concentrations of fibrinogen	NIH3T3 murine fibroblasts	Lipoplexes	Highest luciferase expression (luminescence/10^4^ cells) was 550 for concentrations of 25 mg/mL. Lowest expression was 200, for fibrinogen concentrations of 50 mg/mL.	[18]
Hyaluronic acid hydrogels functionalised with RGD peptides	Murine MSCs	PEI polyplexes	RGD concentration of 100 μM resulted in a cumulative luciferase expression (luminescence) ∼ 6 × 10^5^. RGD concentrations of 10 μM and 400 μM resulted in a cumulative luciferase expression (luminescence) ∼ 4.5 × 10^5^	[161]

Different natural biomaterials, including collagen [14] and fibronectin [187], were also used. Among fibronectin fragments, the ones preferentially activating the *α*3/*α*5*β*1 over the *α*v*β*3 led to significantly higher transfection, in spite of a lower cell spreading [185]. In one study, surfaces coated with fibronectin, with ECM gels or with collagen IV increased murine MSC spreading and transfection efficiency, compared to uncoated surfaces. Interestingly, despite showing the highest level of polyplex internalisation, surfaces coated with collagen I decreased transfection efficiency [160]. This was attributable to reduced intracellular trafficking of the internalised complexes. Indeed, interaction with fibronectin—but not with collagen I—activated the Rho proteins RhoA, Cdc42 and Rac1. On the contrary, inactivation of Rho decreased transgene expression in fibronectin-coated surfaces, by more than 90% [187].

Addition of cell ligands over a certain threshold may not lead to any additional positive effect: rather an optimal intermediate concentration needs to be found. For instance, hyaluronic acid hydrogels functionalised with 100 μM of RGD peptides led to higher transfection efficiency, than hydrogels functionalised with 10 μM and 400 μM [161]. Similarly, fibrinogen concentration of 25 mg/mL was more efficient than concentrations of 10 or 50 mg/mL [18].

However, ECM is involved in several processes, such as cell adhesion, spreading, internalisation of DNA and even cytoplasmic transport. Thereby, assessing the effect of ECM mimetic results is challenging. Furthermore, scaffolds with different amounts of cell adhesion ligands may also have different stiffness, allowing a different DNA diffusion, DNA release or cellular infiltration. A certain variability can also be observed depending on cell type, with a study showing that different concentrations of fibrinogen were needed to transfect murine fibroblasts or human embryonic kidney [15]. Finally, the seeding approach (i.e. whether cells are embedded or seeded on top of hydrogels) also modulated the effect of ECM cues [15].

### Biomaterial stiffness

In spite of having traditionally been only investigated for its structural and mechanical role, biomaterial stiffness has recently emerged as a key regulator of several cellular processes, including transfection. (Table 4) The most evident effect is related to biomaterial stability, water content, swelling ratio and thereby DNA diffusion and release [111]. Furthermore, be they seeded on a substrate [192, 193] or embedded in 3D structures [9, 188], cells respond to variable stiffness by rearranging their cytoskeleton. Increased substrate stiffness caused reinforcement and acquisition of orientational order of actin stress fibers [190, 193], which in turn influence cell polarity and cell shape [192]. Actin fibres furthermore colocalise with caveolae and regulate endocytosis [34]. Stiffer matrices also activated *β*1-integrin [194, 195] and FAK [188, 190, 194–196], which are involved in contractile force [194, 195]. It was also reported a correlation between substrate stiffness and expression of RhoA [197], which is the main regulator of stress fibres [34], and is involved in polyplex transport within the cytoplasm [93, 187]. Differences in cell morphology and actin cytoskeletal rearrangement could be observed even when comparing cells seeded on tissue plastic dishes, or on the softer substrate polydimethylsiloxane (PDMS) [142]. By further varying concentrations of a curing agent, a study fabricated PDMS substrates with variable stiffness (from 50 kPa to 1 MPa), to grow murine chondrocytes [197, 198]. It showed that lower stiffness correlated with reduced intercellular connections and gap junctions, reduced cell area and absence of highly organised paralleled actin fibres [198].

**Table 4:** Biomaterial stiffness and gene delivery. Research papers assessing the effect of biomaterial stiffness on non-viral-mediated gene delivery

Biomaterial	Cell type	Transfection system	Change in transfection efficiency	Ref.
MAP hyaluronic acid hydrogels of different stiffness	Human dermal fibroblasts	jetPEI®	Stiffer hydrogels resulted in a cumulative transgene expression (luminescence) of 2 × 10^7^. Softer hydrogels resulted in a cumulative transgene expression (luminescence) of 1 × 10^7^.	[185]
PLGA porous scaffolds of different stiffness	Human ADSCs	Chitosan polyplexes	Transgene expression was 2-fold higher in stiffer scaffolds, compared to soft ones.	[188]
Alginate gels of different stiffness, conjugated with RGDSP peptides substrates	murine preosteoblasts	PEI polyplexes	Transgene expression 4 times higher in stiffer substrates compared to soft ones.	[189]
Gelatin-coated silicone hydrogels of different stiffness	Human ADSCs	Nanolipoplexes added in the culture medium	Nanolipoplexes were more efficiently internalised in stiffer substrates (∼70 ng internalised per stiff scaffold, compared to 60 ng per soft scaffolds)	[190]
PEGDA hydrogels of different stiffness	NIH3T3 murine fibroblasts, BMSCs, and C2C12 skeletal myoblasts	PEI polyplexes	Stiffer hydrogels led to higher transgene production: 700 ng in stiff hydrogels and 400 ng in soft hydrogels, for fibroblasts. 200 ng in stiff hydrogels and 100 ng in soft hydrogels, for BMSCs. 100 ng in stiff hydrogels and ∼ 50 ng in soft hydrogels, for C2C21	[191]
Hyaluronic acid hydrogels of different stiffness	Murine MSCs	PEI polyplexes	Soft hydrogels led to a higher transgene expression (luminescence ∼ 5 × 10^5^), compared to stiff ones (luminescence ∼ 1 × 10^5^)	[161]
3D collagen-I-alginate hydrogels, of different stiffness and collagen: alginate ratios	Human BMSCs	3D-FectIN™ (OZ Biosciences)	Stiff hydrogels led to a 5,000-fold increase in transgene expression, compared to cells transfected using 2D systems. At collagen: alginate ratio (1:2), transgene expression in stiff hydrogels was 20-fold higher, than in soft ones.	[14]

Biomaterials with tuneable stiffness are generally prepared by either crosslinking the polymer or by varying its concentration. However, if natural biomaterials are used, the former approach may reduce the number of available cell-binding sites [199–201], whereas the latter alters the cell ligand density. Thus, inert polymers can be mixed with natural, or RGD-functionalised ones [202]. For instance, hydrogels containing alginate (i.e. inert component) and collagen can be used. Alginate self-polymerises after the addition of calcium carbonate [9]: by varying the concentration of the latter, stiffness can be controlled [202].

Cells can be either seeded on top of substrates or encapsulated in gels of different stiffness. In the first case, higher stiffness generally correlated with increased transfection [185, 188, 189]. For instance, in PLGA porous scaffold, higher stiffness (modulus of 10,000 Pa, compared to 1000 Pa) increased transfection efficiency at day 5 [188]. Human ADSCs, seeded on stiffer (32 KPa) gelatin-coated silicone hydrogels, were more efficiently transfected, compared to cells on soft (0.5 kPa) surfaces. They also showed increased actin stress fibres and higher expression of caveolin-mediated genes filamin A, caveolin 1 (Cav-1) and integrin *β*1 [190]. Murine fibroblasts, BMSCs and myoblasts were more efficiently transfected, if seeded on poly(ethylene glycol) diacrylate (PEGDA) surfaces with higher elastic modulus (670 kPa, compared to 10 and 320 kPa) and lower swelling ratio (4.5, compared to 16 and 10, respectively) [191]. Higher transfection was reported for human dermal fibroblasts seeded on MAP gels of high (970 MPa), compared to low (377 and 277 MPa) stiffness [185].

An increase in transfection at increased stiffness could be explained by the fact that stiffer matrices increase cellular adhesion point density [203], activating FAKs [188, 190, 194–196] and facilitating actin stress fibre formation [198, 203]. Additionally, one study attributed their promoting effect to an increase in cell proliferation [189]. As a matter of fact, proliferating cells are more receptive to transfection [204]. When grown on stiffer substrates (alginate conjugated with RGDSP peptides), murine preosteoblasts proliferated faster and were more efficiently transfected with polyplexes. Inhibition of proliferation effectively decreased transfection efficiency [189].

With respect to cell embedding, discordant results were reported, with higher stiffness not necessarily correlating with increased transfection. For instance, one study showed that softer HA hydrogels (300 Pa) resulted in higher transfection efficiency, compared to stiffer ones (800 Pa) [101]. Similarly, softer HA hydrogels (100 and 260 Pa storage moduli) were degraded faster by collagenase I and hyaluronidase, they were less stable in PBS and released more DNA polyplexes, compared to stiffer ones (1730 and 1360 and 839 Pa). They also led to higher cell spreading and transfection efficiency, measured 2, 4 and 6 days after seeding [161]. The reason could be that high stiffness also resulted in longer degradation rates, DNA release profiles and distinct transgene expression kinetics. Furthermore, stiffer hydrogels may limit cellular infiltration and migration, yet transfected cells are predominantly the ones migrating within the biomaterial [162]. Clathrin-mediated vesicles are indeed implicated in cell migration [12, 13], with an increased rate of endocytosis at the trailing edge, away from the lagging edge [205]. Additionally, excessive tension, resulting from substrates with high stiffness [206], might alter and stall clathrin-mediated endocytosis [207, 208]. Having said this, no clear consensus has, in fact, been reached as yet: in one study, stiff collagen I /alginate hydrogels (storage modulus of 1500 Pa) led to a transfection efficacy of encapsulated human MSCs, ten times higher than soft ones (150 Pa) [14]. Another study, although did not measure the modulus, found that intermediate concentrations of fibrinogen (25 mg/mL, compared to 10 or 50 mg/ mL) led to higher transfection of entrapped murine fibroblasts [18]. Yet, differences might also be attributed to the different cell ligand densities. Finally, another study showed that stiffer HA hydrogels (∼ 1200 compared to ∼ 600 Pa) led to higher transfection efficiency. Yet, a different amount of loaded nioplexes (0.12 μg/ μL in the stiffer gels and 0.055 μg/μL in the softer ones) may also explain the difference observed [126].

In general, in spite of several studies assessing the biological effect of substrate stiffness [194, 195, 209–216], cytoskeletal trafficking/structural changes in cells encapsulated in biomaterials of different stiffness has been scarcely investigated, with no consensus having been reached as yet. Further research on this direction is limited by the fact that biomaterial stiffness influences several other different processes, including biomaterial degradation rate, cellular infiltration, migration and proliferation rate. Furthermore, whilst available research studies only compared the efficacy of two or three different fabrication systems, comprehensive studies of the interaction between different parameters are still needed. Thereby, potential mechanisms to increase DNA internalisation and nuclear trafficking by controlling biomaterial stiffness still remain to be determined.

### Mechanical loading

In spite of discordances in terms of optimal loading regimens, it is recognised that mechanical compression modulates gene transfection, as it induces structural changes in the cytoskeleton. (Table 5) In A549 cells, equibiaxial stretching applied immediately after transfection and for as little as 30 min, resulted in a 10-fold increase in gene transfer [223]. Similarly, A549 cells, subjected to equibiaxial stretching on silastic membranes, reorganised their cytoplasm, appeared more rounded than their unstretched counterparts and were more efficiently transfected via electroporation [222]. By using a dielectric elastomer actuator (DEA)-based stimulation bioreactors to generate tensile and contractile stress, in A549 cells, optimised cell compression (in terms of frequency and duty cycle) had a promoting effect, whereas different stretching regimens showed an inhibitory effect on transfection [38]. Although molecular mechanisms were not investigated, the decrease of gene transfection in stretched—as opposed to compressed—cells could be attributed to caveola flattening and disassembly, which was proven to be a consequence of excessive mechanical tension [224, 225]. This effect is probably mediated by the tyrosine kinase Src [225] and by the mDia1, a regulator of actin polymerisation [226, 227], which also acts as caveolar domain organisation [226]. Furthermore, excessive membrane tension might have prevented budding of clathrin-coated vesicles and slowed down their dynamics [207, 228].

**Table 5:** Mechanical stimulation and gene delivery. Research papers assessing the effect of different mechanical loading regimens on non-viral-mediated gene delivery

Type of mechanical stimulation	Cell type	Transfection system	Change in transfection efficiency	Ref.
Uniaxial cyclic stretch (0-10%, 0.5 Hz, 60 min), equiaxial cyclic stretch (5%, 05. Hz, 15 min) and shear stress (0.5 3 degrees torsion, 0.5 Hz, 15 min)	Murine primary lung epithelial cells on silicone membranes	Cationic polymer TurboFect™ (ThermoFisher) or naked DNA added after loading	Independently on the type of stimulus, optimal loading regimens increased transfection efficiency, compared to less intense or more intense regimens (in terms of % time, loading time and frequency), for both polyplexes and naked DNA. Using TurboFect™, % transfection was ∼ 63% for mechanically loaded samples and ∼ 5%, for non-loaded control.	[217]
Sine Wave Generator, 100 Hz, 10 V amplitude for 4 min	Myelogenous leukaemia cell line K562 in suspension	Naked siRNA added during loading	10-time fold increase in transfection efficiency in mechanically loaded samples, compared to non-loaded control	[218]
Uniaxial cyclic stretch, equiaxial cyclic stretch and shear stress bioreactors	Dendritic cells and mesenchymal stem cells on silicone membranes	TurboFect™ or Lipofectamine™ added after loading	Equiaxial cyclic stretch loading combined with Lipofectamine™ led to the highest transfection efficiency (60.21% for MSCs, and 65.06% for dendritic cells)	[219]
Uniaxial stretching (10%, 0.5 Hz, 30 min)	HEK 293 on silicone membranes	Naked DNA added after loading	Uniaxial stretching allowed internalisation of naked DNA, resulting in transfection efficiency of 47%. No results shown for non-loaded controls	[220]
Stretching or compression (compressing ratio 2%, 4%, 6% and stretching ratio 4%, 8%, 12%, 30, 60 and 100% duty cycle, 2, 10, 100, 1000 mHz, for 10 min)	A549 cells on silicone membranes	Lipofectamine™, added after mechanical loading	A 30% increase in transfection efficiency was observed for compressed samples (duty ratios 60% and 100%, loading frequency 2 mHz, compression ratio 4% and 6), compared to non-loaded control. A 40% decrease in transfection efficiency was observed in stretched samples, compared to non-loaded control.	[38]
Ultrasound-induced mechanical stress (50 pulses of 250 *μ*s in duration and 600 V in amplitude), combined with high electric field (electroporation)	In vivo rabbit retinas and in vitro chorioallantoic membrane	Naked DNA added during stimulation	1000-fold increase in photons/s indicating a higher expression of the marker gene luciferase in samples subjected to ultrasound and electroporation treatments, compared to samples subjected to electroporation alone.	[221]
Equibiaxial cyclic stretch (10% area stretch, 50% duty cycle, 0.5 Hz, time variable from 30 min to 24 h)	A549 on Pronectins™-treated plates	Electroporation of naked DNA, performed before mechanical loading	Twenty-four hours of cyclic stretch induced a onefold to sixfold increase in transfection, compared to non-loaded control	[222]
Equibiaxial stretch, either continuous or cyclic (10% area stretch, 10% duty cycle, 1 Hz, either 30 min or 24 h)	A549 on laminin-coated plates	Naked DNA, or lipoplexes composed of Lipofectin™ (Thermofisher) or electroporation. Transfection performed before and/or after loading	If performed before transfection, mechanical loading had no effect. If performed after transfection, cyclic—but not continuous—stretching significantly increased transfection efficiency for both Lipofectin™ and electroporation but had no effect on naked DNA delivery system. Specifically, 30 min of stretching was sufficient to achieve a 2-fold (for Lipofectin™) or even a 10-fold (for electroporation) increase in transgene expression.	[223]

Apart from inducing cytoskeletal remodelling, when performed during [218], or immediately after DNA administration [217], mechanical stimulation, by inducing pore formation to the cell membranes, facilitated DNA entering [217, 218, 229]. Nevertheless, such mechanisms were not largely investigated, nor has a consensus on the most efficient loading parameters been reached as yet. For instance, whilst uniaxial stretching decreased transfection of A549 cells [38], it did show a beneficial effect on murine primary lung epithelial cells [217] and HEK293 [220]. (Table 2) Furthermore, it is understood that optimal mechanical regimens increase cytoskeletal trafficking, but whether mechanical loading has any role in diverting endocytic pathways has not been investigated as yet, with only one suggesting a predominance of clathrin-coated vesicles, over caveolae and micropinocytosis for mechanically stimulated cells [38].

## Conclusions and future directions

By combining biomaterials with gene therapy, it is possible to create tailored non-viral delivery systems, suitable for various applications. Different biomaterial fabrication strategies (i.e. DNA immobilisation, encapsulation or surface coating) lead to distinct temporal patterns of DNA release and transgene expression. The addition of mechanical or topographical cues, that facilitate cellular spreading, can further increase gene delivery. By incorporating ECM mimetics, synthetic peptides or through distinct chemical modifications, it is possible to target specific cell types or to create responsive systems with precise controlled features. Certain biomaterials may furthermore possess a therapeutic potential themselves, which can be combined with that of gene therapy [230]. Nevertheless, there still are many outstanding questions surrounding biomaterial-guided gene delivery. Firstly, endocytic pathways vary depending on cell type and DNA complex, and they still need to be fully characterised. Furthermore, nor potential mechanisms to escape the lysosomal network or mechanism of action of CPP are fully understood. It is not clear to which extent caveola-mediated endocytosis allows DNA to escape lysosomal trafficking, and eventually, the most effective strategies to divert DNA towards caveolar vesicles are to be unravelled. Caveolae closely co-align with actin stress fibres [34], and switch from clathrin to caveola-mediated endocytosis has been attributed to a change from actin polymerisation to depolymerisation [231], as Cav-1 appears to be dragged by depolymerising actin filaments and then concentrate with resulting actin patches [34, 226, 232]. Thereby, diversion of DNA trafficking towards caveolar vesicles could theoretically be facilitated by 3D embedding culture systems [91–93] and be further modulated by incorporating topographic cues [148] or ECM mimetics [187], which, by increasing cell adhesion area, force cells to rearrange actin filaments. Also, substrates with increased stiffness induced structural changes in the cytoskeleton, increased caveola dynamics [190], enhancing gene transfection [185, 188, 189]. Yet, development of robust engineered systems is challenging, as biomaterial mechanical properties influence a plethora of other different processes, including DNA diffusion and release, biomaterial degradation, cellular infiltration, migration and proliferation rate. All these processes may in turn affect transgene expression. This is even more evident when cells are embedded, as opposed to seeded, on the biomaterials. For embedding systems, not even a general consensus on whether increased stiffness facilitates [14] or stalls [101, 161] gene delivery has been reached as yet.

Mechanical loading also induces actin depolymerisation [233, 234] and structural changes in the microfilament and microtubule network [222]. The loading-induced increase in gene delivery was effectively abolished by cytoskeletal-stabilising compounds [222]. Yet, despite having been proven, the beneficial effect of mechanical loading has not been extensively studied. It is not clear whether it was a different stimulus (e.g. uniaxial stretching versus compression [38]/equiaxial stretching) [219] or rather an excessive mechanical tension (independent on the type of loading) [207, 224, 225, 228], which under certain conditions, led to caveola and clathrin vesicle disassembling.

Furthermore, the correlation between transgene expression and DNA endocytosis or nuclear entry is not always straightforward, with for instance collagen I coating showing a higher level of polyplex internalisation, but reduced intracellular trafficking and transgene expression, compared to fibronectin [160]. Similarly, compared to PLL, PEI polyplexes showed a lower cytoplasmic uptake, but a higher percentage of nuclear uptake [76]. Although this was beyond the scope of this review, it is noteworthy to mention that the in vivo fate of biomaterial-mediated gene delivery systems is also influenced by many other parameters, including eventual interaction with plasma proteins [235], or potential triggering of an innate immune response [236]. Only a better understanding of these processes may allow to the creation of novel robust engineered systems, potentially opening up a whole new area of biomaterial-guided gene delivery for non-viral systems.

## Data Availability

Not applicable.

## Abbreviations

ADSCs: Adipose-derived stem cells
BMSCs: Bone marrow mesenchymal stem cells
CPP: Cell-penetrating peptide
DEA: Dielectric elastomer actuator
DOPE: 1,2-Dioleoyl-sn-glycero-3-phosphoethanolamine (DOPE)
DOTAP: 1,2-Dioleoyl-3-trimethylammonium propane
DOTMA: 1,2-Di-O-octadecenyl-3-trimethylammonium propane
DOPC: dioleoylphosphocholine
ECM: Extracellular matrix
ER: Endoplasmic reticulum
FAK: Focal adhesion kinases
GGT: Gamma-glutamyl transpeptidase
HA: Hyaluronic acid
hAM-MSCs: Human amniotic membrane-derived mesenchymal stem cells
MAP: Microporous annealed particle
mDia1: Diaphanous-related formin-1
mESCs: Mouse embryonic stem cells
MMP: Matrix metalloproteinase
MSCs: Mesenchymal stem cells
MTOC: microtubule-organising centre
NPC: Nuclear pore complexes
OPF: Oligo (polyethylene glycol) fumarate
PAA: Poly(acrylic) acid
PAH: Poly(allylamine hydrochloride)
PAMAM: Polyamidoamine
PBS: Poly(beta-amino ester)
PD: Polydopamine
PDDA: Polydiallyldimethylammonium
PDMS: Polydimethylsiloxane
PEGDA: Poly(ethylene glycol) diacrylate
PEI: polyethylenimine
PLGA: Poly(lactic-co-glycolic acid)
PLL: Polylysine
PMMA: Poly(methyl methacrylate)
pnAC: Perinuclear actin cap
PTD: Protein transduction domain
RGD: Arginine-glycine-aspartate
siNWAs: Silicon nanowires
SMD: Surface-mediated gene delivery
γ-PGA: Poly-γ glutamic acid
